# Langerhans cells orchestrate apoptosis of DNA‐damaged keratinocytes upon high‐dose UVB skin exposure

**DOI:** 10.1002/eji.202451020

**Published:** 2024-09-17

**Authors:** Daniela Ortner, Helen Strandt, Christoph H. Tripp, Sarah Spoeck, Athanasios Seretis, Florian Hornsteiner, Sophie Dieckmann, Matthias Schmuth, Patrizia Stoitzner

**Affiliations:** ^1^ Department of Dermatology, Venereology & Allergology Medical University of Innsbruck Innsbruck Austria; ^2^ Division of Developmental Immunology Medical University of Innsbruck Innsbruck Austria; ^3^ Research Institute for Biomedical Ageing Research University of Innsbruck Innsbruck Austria

**Keywords:** Apoptosis, Langerhans cells, Neutrophils, Skin inflammation, UVB‐induced sunburn

## Abstract

Ultraviolet (UV) irradiation of the skin causes mutations that can promote the development of melanoma and nonmelanoma skin cancer. High‐dose UVB exposure triggers a vigorous skin reaction characterized by inflammation resulting in acute sunburn. This response includes the formation of sunburn cells and keratinocytes (KC) undergoing programmed cell death (apoptosis) when repair mechanisms of DNA damage are inadequate. The primary objective of this research was to clarify the involvement of Langerhans cells (LC) in the development of acute sunburn following intense UVB skin irradiation. To address this, we subjected the dorsal skin of mice to a single high‐dose UVB exposure and analyzed the immediate immune response occurring within the skin tissue. Acute sunburn triggered an activation of LC, coinciding with a rapid influx of neutrophils that produced TNF‐α. Furthermore, our investigation unveiled a marked increase in DNA‐damaged KC and the subsequent induction of apoptosis in these cells. Importantly, we demonstrate a crucial link between the inflammatory cascade, the initiation of apoptosis in DNA‐damaged KC, and the presence of LC in the skin. LC were observed to modulate the chemokine response in the skin following exposure to UVB, thereby affecting the trafficking of neutrophils. Skin lacking LC revealed diminished inflammation, contained fewer TNF‐α‐producing neutrophils, and due to the prevention of apoptosis induction, a lingering population of DNA‐damaged KC, presumably carrying the risk of enduring genomic alterations. In summary, our results underscore the pivotal role of LC in preserving the homeostasis of UVB‐irradiated skin. These findings contribute to a deeper understanding of the intricate mechanisms underlying acute sunburn responses and their implications for UV‐induced skin cancer.

## Graphical abstract

Our study reveals that high‐dose UVB exposure stimulates Langerhans cells to release chemokines that recruit neutrophils and induce TNF‐α‐mediated apoptosis in DNA‐damaged keratinocytes. Without Langerhans cells, fewer neutrophils are recruited, leading to an accumulation of DNA‐damaged cells and a higher risk of skin cancer due to increased mutations and potential cell transformation.

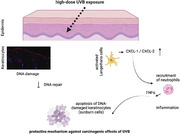

## Introduction

Ultraviolet (UV)‐radiation (UVR) is known to be the predominant cause of human skin cancer since UVR‐induced DNA damage plays a key role in the initiation phase of skin carcinogenesis. Mutations introduced by UVR can lead to melanoma and nonmelanoma skin cancer, for example, basal cell carcinoma and squamous cell carcinoma [1, 2]. UVR causes DNA damage and UV‐specific mutations by direct mechanisms, for example, the introduction of photolesions like cyclobutane pyrimidine dimers (CPDs) and by indirect pathways leading to the formation of reactive oxygen species (ROS) and cellular oxidative stress [3].

UVB with a wavelength of 280–315 nm is known to elicit the main carcinogenic effects in the skin [4, 5, 6]. UVB‐induced signals alter cell physiology, mediate cell cycle arrest, activate DNA repair, and induce apoptosis if the damage is excessive [6, 7]. Sunburn cells (SBC) are KC that undergo apoptosis upon UVB irradiation causing irreparable and severe DNA damage. Programmed cell death of DNA‐damaged cells is required to prevent the introduction of mutations and subsequent carcinogenic effects upon UV radiation. A disruption in the apoptotic process consequently elevates the susceptibility to develop skin cancer [7] as DNA damage with its mutagenic characteristics triggers pro‐oncogenes or the deactivation of tumor suppressor genes [6].

The severity of sunburn is dependent on the intensity of UVB radiation and is recognized as UVB‐induced cutaneous inflammation. Several studies could demonstrate that UVB induces the production of cytokines in the skin including IL‐1, IL‐6, IL‐8, IL‐10, granulocyte‐macrophage colony‐stimulating factor, and TNF‐α [8]. These cytokines ultimately lead to inflammation but in the case of TNF‐α also to the induction of apoptosis of damaged KC, both protective mechanisms to prevent DNA damage upon intense UVB irradiation [9, 10, 11].

The inflammatory response is a defense mechanism and skin inflammation is promptly triggered after epidermal damage inflicted by UVB irradiation [12]. Neutrophils represent the predominant white blood cells in the circulation and are quickly mobilized to sites of infection and inflammation [13], including skin exposed to UV light [14].

Langerhans cells (LC) were found to be able to resist DNA damage induced by UV light (notably UVC) with their ability to rapidly repair DNA double‐strand breaks [15]. Further, LC are known for their close association with epidermal KC; however, the early immunological processes and the specific role of LC within the skin prior to their migration upon UVB exposure remain poorly defined, as previous research has predominantly investigated immune responses taking place in skin‐draining lymph nodes [16, 17, 18, 19]. Few studies have explored the function of LC within the skin directly after UVB treatment. One study suggested a potential pro‐carcinogenic role for LC in the context of UVB exposure [20], while another asserted that LC might mediate protection against UVB‐induced apoptosis in KC [21]. Interpreting these findings warrants careful consideration because huLangerin‐DTA mice [22], which lack LC from birth, exhibit alterations in the skin microenvironment [23] and the development of the skin's immune system [24]. By employing an inducible LC depletion mouse model (huLangerin‐DTR mice) [25], we highlight a previously unprecedented function of LC during the initial stages of acute sunburn. Following exposure to a single high dose of UVB radiation, the skin contained a prominent population of activated LC, essential for the recruitment of TNF‐α‐producing neutrophils through the release of chemokines. Furthermore, LC‐deficient skin presented with a notable decrease in the formation of SBC and a significant decline in the initiation of apoptosis in DNA‐damaged KC. In summary, our study uncovers a previously undisclosed function of LC, emphasizing their essential role in the initial inflammatory response following UVB exposure to the skin.

## Materials and methods

### Mice

Breeding pairs for C57BL/6N mice were purchased from Charles River Laboratories. huLangerin‐DTR mice [25] were kindly provided by Angelika Sales from the University of Salzburg. Experimental mice were bred and housed in the Department of Dermatology, Venereology, and Allergology at the Medical University of Innsbruck. Female, 7–12‐week‐old mice, were used for experiments throughout the study. All animal experimental protocols were approved by the Austrian Federal Ministry of Science and Research (66.011/0204‐WF/V/3b/2016 and 2020‐0.827.313) and performed according to institutional guidelines.

### Skin irradiation

The back skin of mice was shaved followed by depilatory cream (Veet) application 2 days before UVB irradiation. 7–12‐week‐old C57BL/6 and huLangerin‐DTR mice were irradiated with a single high‐dose of UVB (1000 J/m^2^ = 1 kJ/m^2^ = 1 kW/m^2^) in an Opsytec Dr. Grobel BS‐02 irradiation chamber. The irradiation chamber was equipped with one spectral region, UV‐B λ = 280–315 nm. The radiation dose was measured with calibrated sensors and controlled using a UV‐MAT radiation controller. Skin samples were harvested at different time point after UVB, respectively.

### Flow cytometry analysis

The back skin of mice was processed as described previously [26]. All staining steps for flow cytometry were performed for 15 min at 4°C unless stated otherwise. Nonspecific Fc‐receptor‐mediated antibody binding was blocked using an anti‐CD16/32 antibody (clone 2.4G2, TONBO Biosciences) incubated for 15 min. For the exclusion of dead cells, samples were stained with eFluor‐780 fixable viability dye (eBioscience), prior to any other staining step. For intracellular staining, the cells were fixed and permeabilized using the BD Biosciences Cytofix/Cytoperm kit according to the manufacturer's protocol. All analyses were performed on a CytoFLEX S (Beckman Coulter Life Sciences). See the antibody list in Supporting Information Table S1.

### RNA isolation and real‐time quantitative PCR

Total RNA was extracted using TRIzol Reagent (Gibco) according to the manufacturer's instructions. RNA integrity was analyzed by electrophoresis on 1.5% agarose gels (Sigma‐Aldrich) after sample preparation in 2 × RNA Loading Dye (Thermo Fisher Scientific). For RT‐qPCR, genomic DNA was removed from total RNA with the RapidOut DNA removal kit (Thermo Fisher Scientific Inc.) and reverse‐transcribed into cDNA with random hexamers and SuperScriptR II Reverse Transcriptase (Thermo Fisher Scientific Inc.) according to the kit's instructions. qPCR analysis was performed on a BioRad CFX96 using Brilliant III Ultra‐Fast qPCR and RT‐qPCR Master Mix (Agilent technologies). Sequences for probes and primers specific for mouse TATA‐binding protein were selected by Primer Express software (Thermo Fisher Scientific Inc.) and synthesized by Microsynth. Other probes and primers were purchased from Thermo Fisher Scientific Inc., see Supporting Information Table S2.

### Histological and immunofluorescence analysis

Mouse skin was fixed in 4% formaldehyde and embedded in paraffin. Sections of 3 µm thickness were obtained and stained with H&E for routine histology. Sunburn cell formation was evaluated on H&E sections, identified by their typical histologic features [27]. The number of SBC was counted among 10^2^ keratinocytes on 4 skin sections per mouse, 3 mice per timepoint. Analysis was performed using an Olympus BH‐2 light microscope (Olympus) equipped with a ProgRes C10plus camera and ProgRes CapturePro 2.8.8 image analysis software (Jenoptik). For immunofluorescence, sections were deparaffinized, and antigen retrieval was facilitated using 10 mM citrate buffer containing 0.5% Tween (pH 6.0). Sections were stained with an anti‐Thymine Dimer antibody [H3] (ab10347, Abcam) overnight at 4°C, followed by the secondary antibody goat anti‐mouse Alexa‐Fluor568 (Invitrogen) for 1 h at room temperature. DAPI (Thermo Fisher Scientific Inc.) was used to counterstain cell nuclei. CPD^+^ cells were counted on 4 skin sections per mouse, 3 mice per group, and visualized using an Olympus BX60 epifluorescence microscope.

### Cell depletion and TNF‐α neutralization

C57BL/6 or huLangerin‐DTR mice were injected intraperitoneally (i.p.) with 100 µg of anti‐TNF‐α mAb (clone XT‐22, Epirus Biopharmaceuticals) or 300 µg of anti‐Gr‐1 mAb (clone RB6‐8C5, Epirus Biopharmaceuticals) 1 day before and on the day of UVB skin irradiation. For LC depletion, huLangerin‐DTR mice were injected i.p. with 0.5 µg of diphtheria toxin (DT) 2 days before treatment.

### Statistical analysis

All graphs display mean ± SEM. Statistical analysis was performed using GraphPad Prism 8.0 (GraphPad Software). Unpaired Student's *t*‐test was used to determine the statistical significance of the mean percentages of cell types between groups. A *p*‐value of <0.05 was considered statistically significant (*), <0.01 very significant (**), <0.001 highly significant (***), and <0.0001 extremely significant (****). Error bars represent the SE of the mean. All statistical analyses and graphics were performed using GraphPad Prism V.8.0.

## Results

### High‐dose UVB skin exposure leads to the formation of CPD and apoptosis induction of DNA‐damaged KC

**Figure 1:**
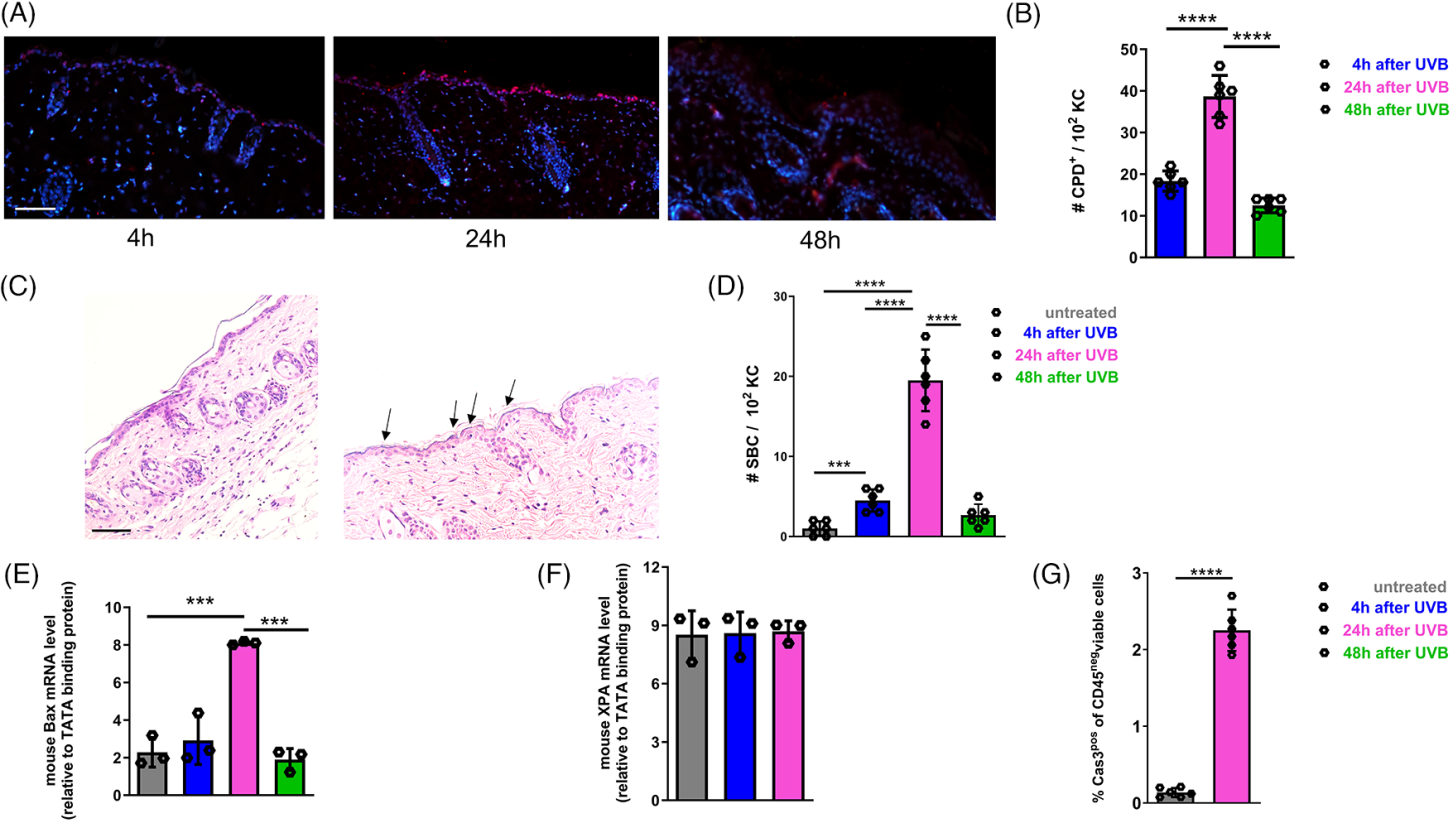
DNA damage and apoptosis induction in KC upon a single high‐dose UVB exposure. A single UVB exposure of 1000 J/m^2^ was administered to the dorsal skin of C57BL/6 mice. (A) Representative images of skin sections for immunofluorescence detection of cyclobutane pyrimidine dimers (CPD)^+^ cells 4, 24, and 48 h upon UVB irradiation. CPD^+^ cells (red fluorescence) are counterstained with nuclei staining by DAPI (blue fluorescence). Scale bar = 100 µm. (B) Quantification of CPD^+^ cells in skin sections at different time points (4, 24, and 48 h) after UVB skin exposure. CPD^+^ cells were counted in 4 areas per section, each data point represents the mean of the 4 areas per individual mouse (*n* = 6 mice, two independent experiments). (C) Representative H&E staining of skin sections 24 h after UVB exposure shows characteristic sunburn cell (SBC) formation indicating apoptotic KC with its pyknotic nucleus and eosinophilic cytoplasm as indicated by black arrows in the epidermis of irradiated skin (right) compared with untreated (healthy) skin (left). (D) The number of SBC^+^ cells was evaluated at different time points (4, 24, and 48 h) after UVB irradiation. SBC were counted in 4 areas per section, each data point represents the mean of the 4 areas per individual mouse (*n* = 6 mice, two independent experiments). (E) mRNA expression level for Bax was analyzed in skin samples at different time points (4, 24, and 48 h) after UVB irradiation (*n* = 3 mice, one experiment). (F) mRNA expression level for XPA was analyzed 4 and 24 h after UVB irradiation and compared with untreated skin of C57BL/6 mice (*n* = 3 mice, one experiment). G) Skin cell suspensions were analyzed by flow cytometry for act‐Cas3^+^ cells, pregated on viable CD45^−^ cells 24 h after UVB irradiation. A summary graph of 6 mice per group is shown from two independent experiments. All graphs show mean ± SEM and each data point represents an individual mouse. *p < 0.05; **p < 0.01; ***p < 0.001; unpaired *t*‐test.

**Figure 2:**
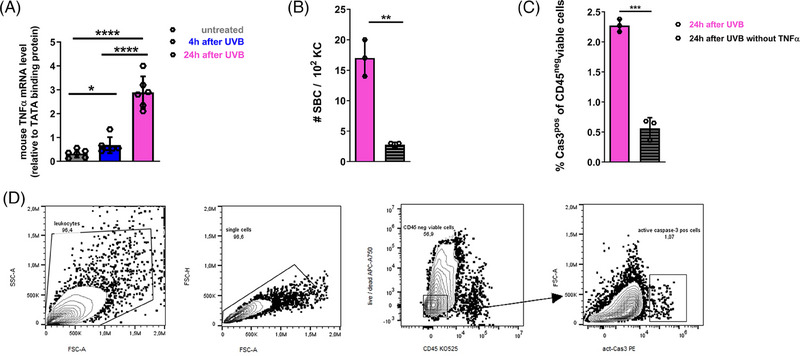
TNF‐α is crucial for apoptosis induction by high‐dose UVB skin irradiation. C57BL/6 mice were irradiated with a single UVB exposure using 1000 J/m^2^ on the back skin. (A) mRNA expression level for TNF‐α in skin was analyzed 4 and 24 h after UVB irradiation (*n* = 6 mice, two independent experiments). (B) Mice were injected intraperitoneally with 100 µg anti‐TNF‐α antibody 1 day before and immediately after UVB irradiation. SBC numbers were evaluated in UVB irradiated skin 24 h after treatment and were counted in 4 areas per section, each data point represents the mean of the 4 areas per individual mouse. (C) The percentages of act‐Cas3^+^CD45^−^ viable cells were determined by flow cytometry in skin 24 h after UVB irradiation. (D) Antibodies that specifically recognize the cell surface marker CD45 and act‐Cas‐3 were used to characterize viable CD45^−^ cells in skin cell suspensions by flow cytometry. Graphs display mean ± SEM, each data point represents an individual mouse, and the summary graph for 3 mice per group analyzed in one experiment is shown for (B) and (C); *p < 0.05; **p <0.01; ***p <0.001; unpaired *t*‐test.

To evaluate the impact of intense UVB exposure on the skin, C57BL/6 mice underwent a single irradiation session with 1000 J/m^2^ on their dorsal skin. Subsequently, skin samples were collected at various time intervals and compared with untreated controls. One instance of high‐dose UVB exposure resulted in DNA damage, leading to the production of CPDs in KC, peaking at 24 h posttreatment and subsequently decreasing after 48 h (Fig. 1A and B). In addition to the formation of CPDs, the treated skin exhibited characteristic SBC formation, representing apoptotic KC (Fig. 1C and D). Consistent with earlier studies involving HaCaT cells exposed to 500 J/m^2^ of UVB, which revealed only 20–31% of DNA‐damaged cells undergoing repair [28, 29], we detected a negligible amount of DNA‐damage repair in our experiments using one high‐dose of 1000 J/m^2^. In this line, the expression of the apoptosis‐associated gene Bax exhibited an increase 24 h post‐UVB irradiation in comparison to untreated skin (Fig. 1E). Furthermore, the assessment of Xeroderma Pigmentosum group A (XPA) protein mRNA expression levels, a crucial regulator in the nucleotide excision repair pathway essential for DNA‐damage repair, revealed comparable XPA mRNA levels in both, irradiated and untreated skin (Fig. 1F) presuming the induction of apoptosis of DNA‐damaged KC. Consistent with this observation, flow cytometry detection of active caspase‐3 (act‐Cas3) positive cells confirmed the induction of apoptosis in KC (Fig. 1G, gating strategy in Fig. 2D).

Overall, our experiments demonstrate that intense UVB exposure induces substantial DNA damage in mouse skin, resulting in the formation of mutagenic DNA photoproducts (CPDs) and subsequent apoptosis of KC.

### TNF‐α is indispensable for apoptosis induction of DNA‐damaged KC

The absorption of UVB results in direct DNA damage [4] and the production of ROS [5]. Several investigations have demonstrated that exposure to UVB induces the production of various cytokines in the skin, such as IL‐1, IL‐6, IL‐8, IL‐10, granulocyte‐macrophage colony‐stimulating factor, and TNF‐α [8, 30]. Among these, TNF‐α stands out as a key player in triggering apoptosis in damaged KC, representing a protective mechanism in response to intense UVB irradiation [9, 10]. To assess the mRNA expression levels of TNF‐α in the skin after exposure to a high dose of UVB, we harvested the back skin of C57BL/6 mice 4 and 24 h posttreatment and compared the samples to untreated skin. As depicted in Fig. 2A, an increase in TNF‐α mRNA expression levels was observed as early as 4 h after UVB skin irradiation when compared with untreated skin, followed by a further significant rise 24 h postirradiation. To assess the necessity of TNF‐α for inducing apoptosis in DNA‐damaged KC, mice were injected intraperitoneally with anti‐TNF‐α monoclonal antibody 1 day prior to and immediately after irradiation. When evaluating SBC numbers, we observed a decline in numbers following TNF‐α neutralization (Fig. 2B). In addition, blocking TNF‐α resulted in significantly reduced percentages of act‐Cas3 positive KC, as determined by flow cytometry analysis of skin cell suspensions 24 h after treatment (Fig. 2C, gating strategy is shown in Fig. 2D). These results provide additional evidence of reduced apoptosis induction in KC in the absence of TNF‐α.

Thus, the overall data are in line with earlier studies [9, 31] confirming an important role for TNF‐α during the formation of SBC reflecting induction of apoptosis of DNA‐damaged KC upon UVB irradiation.

### Activated LC within UVB‐irradiated skin contributes to the production of TNF‐α and the initiation of apoptosis in DNA‐damaged KC

**Figure 3:**
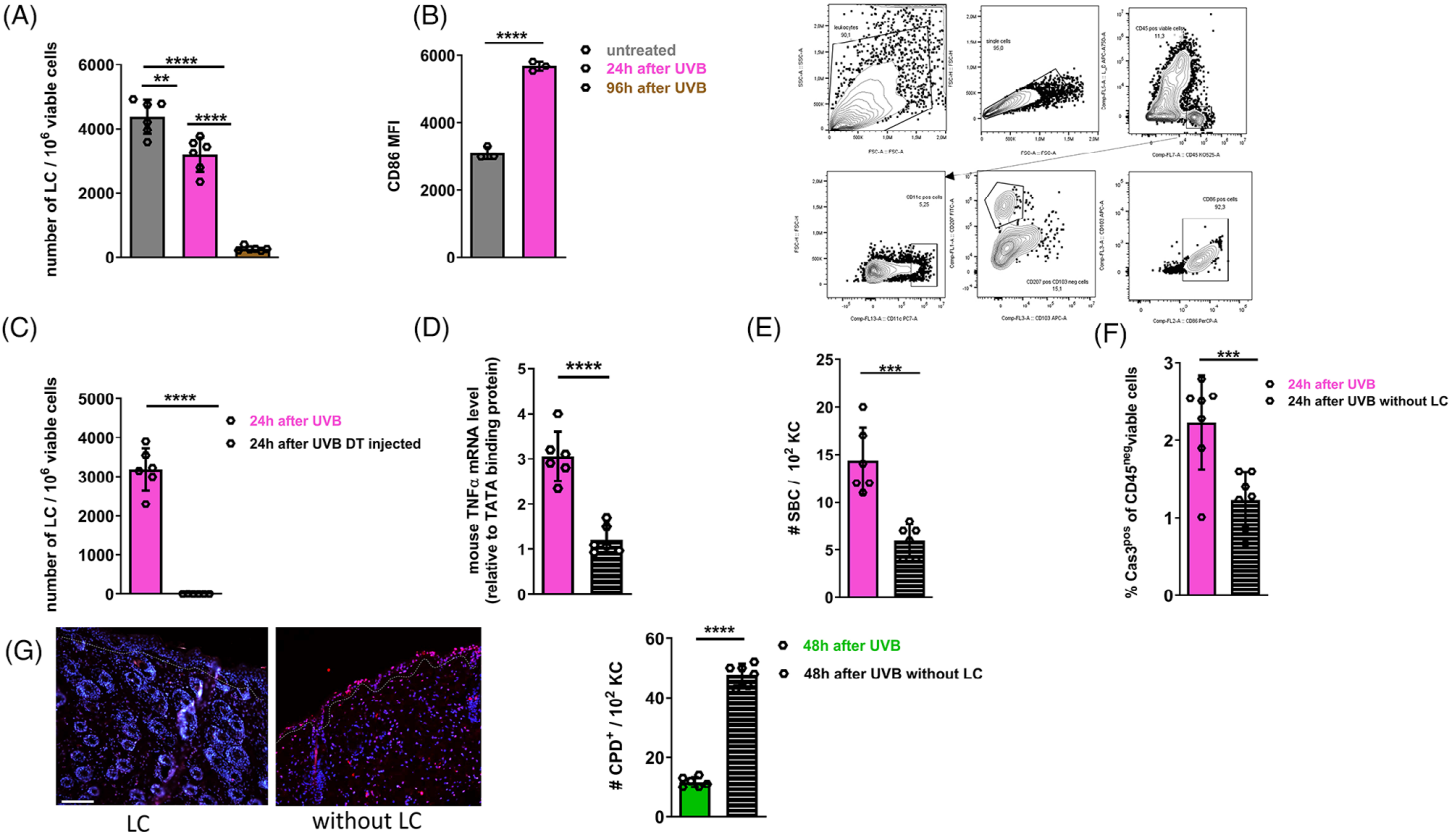
The crucial role of LC during sunburn cell formation and apoptosis induction of KC. C57BL/6 or huLangerin‐DTR mice were irradiated with a single UVB exposure using 1000 J/m^2^ on the back skin. (A) The numbers of CD45^+^CD11c^+^CD103^−^CD207^+^ viable LC were determined by flow cytometry 24 and 96 h after UVB irradiation in C57BL/6 mice (*n* = 6 mice, two independent experiments). (B) LC were identified as CD45^+^CD11c^+^CD103^−^CD207^+^ viable cells. CD86 median fluorescence intensity (MFI) was determined by flow cytometry (*n* = 3 mice, one experiment). Representative flow cytometry plots show the gating strategy for CD86^+^ LC. (C) Skin cell suspensions of huLangerin‐DTR mice injected intraperitoneally with 0.5 µg DT 2 days prior to UVB irradiation were analyzed for the presence of LC 24 h after UVB n by flow cytometry (*n* = 6 mice, two independent experiments). (D) mRNA expression levels for TNF‐α were analyzed 24 h after UVB irradiation comparing the skin of huLangerin‐DTR mice in the presence and absence of LC during UVB irradiation (*n* = 6 mice, two independent experiments). (E) The numbers of SBC were evaluated in the skin of depleted and non‐depleted huLangerin‐DTR mice 24 h after UVB irradiation. SBC were counted in 4 areas per section, and summary graphs for s mice per group from two independent experiments are shown. (F) The percentages of act‐Cas3^+^CD45^−^ viable cells were determined in the skin of depleted and non‐depleted huLangerin‐DTR mice by flow cytometry 24 h after UVB irradiation (*n* = 6 mice, two independent experiments). (G) Representative images of skin sections for immunofluorescence staining of CPD^+^ cells 48 h after UVB irradiation of mice depleted (right) or nondepleted (left) of LC are shown. CPD^+^ cells (red fluorescence) are counterstained with nuclei staining by DAPI (blue fluorescence). Scale bar = 100 µm. (H) Quantification of CPD^+^ cells in skin sections 48 h after UVB skin exposure. CPD^+^ cells were counted in three areas per section, each data point represents the mean of the three areas per individual mouse (*n* = 6 mice, two independent experiments). All graphs display mean ± SEM, each data point represents an individual mouse. *p < 0.05; **p < 0.01; ***p < 0.001; unpaired *t*‐test.

In a previous investigation of chemical carcinogenesis in a mouse skin cancer model we noted a TNF‐α‐dependent recruitment of innate immune cells to the skin during the initiation phase. This recruitment was specifically reliant on the presence of LC in the skin [32]. Hence, we initially compared the numbers of LC in skin exposed to high‐dose UVB irradiation and compared them with untreated skin. We observed a prominent proportion of LC still present in the skin 24 h after treatment, followed by a substantial decline after 96 h (Fig. 3A). Besides LC, other immune cell types were diminished in the skin upon UVB irradiation. Overall numbers of CD11c^+^ DC, macrophages, and dendritic epidermal T cells decreased over 96 h, whereas numbers of neutrophils increased (Supporting Information Fig. S1). Moreover, LC that persisted in UVB‐treated skin after 24 h exhibited signs of activation, evidenced by high CD86 expression (Fig. 3B).

To assess the possible involvement of LC during sunburn induction, LC were selectively eliminated in huLangerin‐DTR mice 2 days prior to UVB exposure by intraperitoneal injection of 0.5 µg of DT (Fig. 3C). Eliminating LC in huLangerin‐DTR mice led to a significant decrease in TNF‐α mRNA expression levels in UVB‐treated skin, observed 24 h after irradiation (Fig. 3D). Besides diminished TNF‐α availability, we detected significantly lower numbers of SBC (Fig. 3E) and act‐Cas3^+^ KC (Fig. 3F) in mice lacking LC during sunburn induction with high‐dose UVB. Furthermore, numbers of DNA‐damaged KC as determined by CPD^+^ cells were significantly elevated 48 h after UVB skin exposure in mice lacking LC (Fig. 3G).

Altogether, LC are essential for enhanced TNF‐α production in the skin following high‐dose UVB exposure and the subsequent induction of apoptosis in DNA‐damaged KC. Skin devoid of LC exhibited a persistent population of DNA‐damaged KC, which likely increases the risk of long‐term genomic alterations.

### LC are required for chemokine‐mediated recruitment of TNF‐α‐producing neutrophils to UVB‐exposed skin

**Figure 4:**
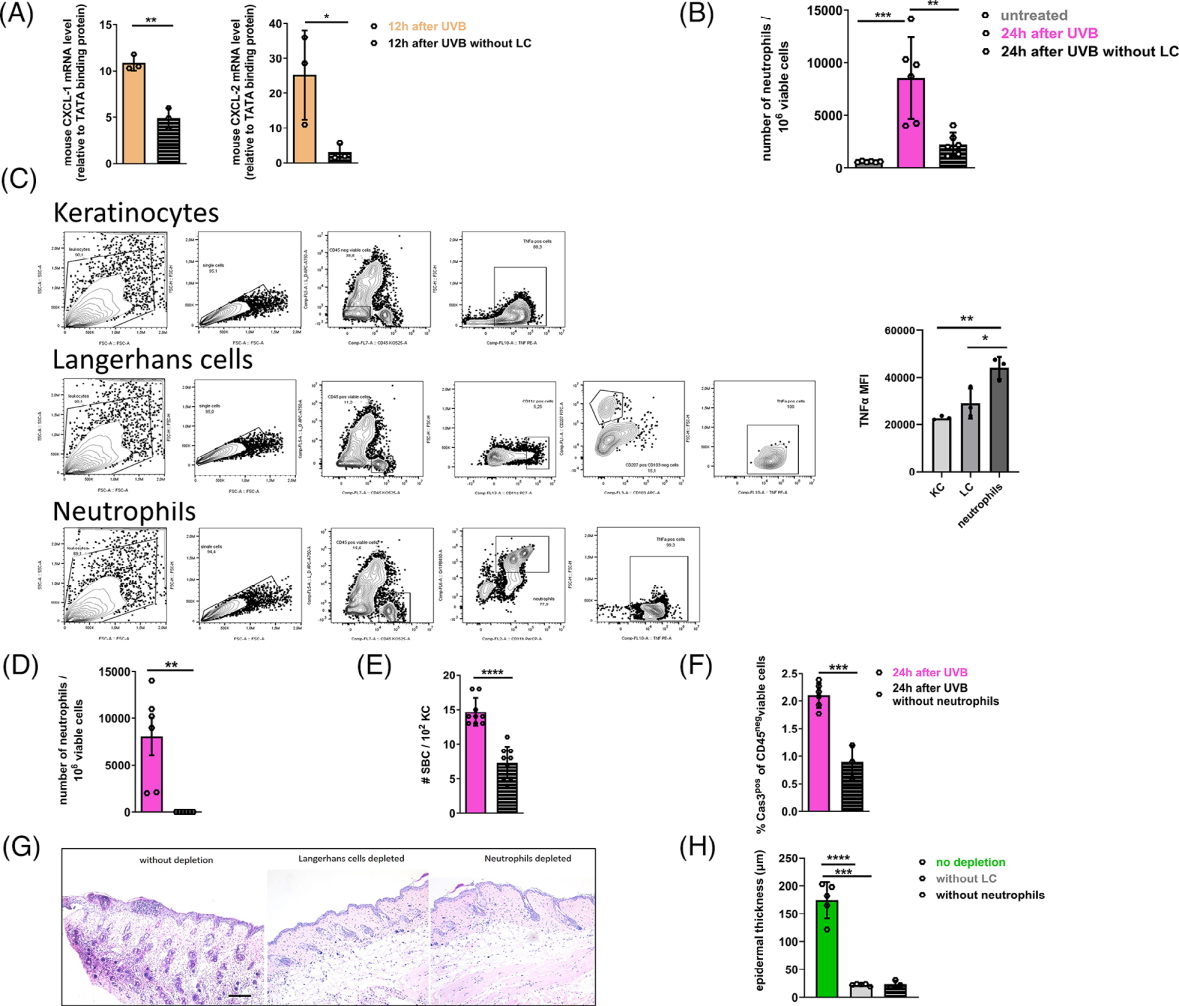
LC are important for the recruitment of TNF‐α‐producing neutrophils to acute sunburn skin. huLangerin‐DTR mice were irradiated with a single UVB exposure using 1000 J/m on the back skin. LC were depleted 2 days before UVB irradiation by intraperitoneal injection of 0.5 µg of DT. (A) The mRNA expression level of CXCL‐1 and CXCL‐2 in the skin of depleted and non‐depleted huLangerin‐DTR mice was analyzed 12 h after UVB treatment; *n* = 3 mice, one experiment. (B) Skin cell suspensions of depleted or non‐depleted huLangerin‐DTR mice were analyzed for the numbers of neutrophils (CD45^+^CD11b^+^Ly6G ^+^ viable cells) 24 h after irradiation by flow cytometry (*n* = 6 mice, two independent experiments). (C) Representative flow cytometry gating strategies for analyzing TNF‐α production in CD45^−^ keratinocytes (KC), Langerin^+^ LC, and Ly6G^+^ neutrophils in the skin 24 h after UVB exposure are shown. The comparison of TNF‐α production by KC, LC, and neutrophils is displayed as the median fluorescence intensity (MFI) by flow cytometry (*n* = 3 mice, one experiment). (D) huLangerin‐DTR mice were injected intraperitoneally with anti‐Gr‐1 or PBS 2 days before and on the day of UVB irradiation. Skin cell suspensions of depleted or non‐depleted huLangerin‐DTR mice were analyzed for the numbers of CD45^+^CD11b^+^Ly6G^+^ viable neutrophils 24 h after UVB irradiation by flow cytometry (*n* = 6 mice, two independent experiments). (E) The number of sunburn cells (SBC) was evaluated in skin 24 h after UVB irradiation. SBC were counted in three areas per section (*n* = 6 mice, two independent experiments). (F) The percentages of act‐Cas3^+^ CD45^−^ viable cells were determined by flow cytometry; *n* = 6 mice, two independent experiments. (G) Representative images of H&E‐stained histological sections displaying epidermal thickening 48 h after UVB exposure comparing the skin of mice not depleted to skin depleted of LC or neutrophils. Scale bars = 100 µm. (H) Epidermal thickness was measured using ImageJ software 48 h following UVB exposure. Three areas of each slide have been examined; each data point represents the mean of the 3 areas per one individual mouse (*n* = 6 mice, two independent experiments). All graphs display mean ± SEM, each data point represents an individual mouse. *p < 0.05; **p < 0.01; ***p < 0.001; unpaired *t*‐test.

To further explore the intricate mechanism by which LC are involved in the early development of sunburn as well as the inflammatory response following high‐dose UVB exposure, we compared the skin of huLangerin‐DTR mice, in which LC were depleted before UVB irradiation, with LC‐intact skin. We first investigated differences in the production of chemokines and indeed skin lacking LC exhibited reduced mRNA expression levels of CXCL‐1 and CXCL‐2, chemokines known to play a crucial role in recruiting neutrophils to the skin [33, 34, 35] (Fig. 4A). Subsequent analysis using flow cytometry revealed a rapid infiltration of neutrophils into UVB‐irradiated skin, a process significantly prevented when LC were depleted (Fig. 4B). Additionally, as determined by flow cytometry analysis, neutrophils were identified as the primary source of TNF‐α in the inflammatory response following a single high‐dose of UVB radiation (Fig. 4C). The depletion of neutrophils by injection of anti‐Gr1 monoclonal antibody (Fig. 4D) led to a significant reduction in the percentages of act‐Cas3^+^ KC (Fig. 4F) as well as decreased SBC numbers (Fig. 4E). Finally, histological examination revealed a significant increase in epidermal thickness and the presence of cellular infiltrate in skin 48 h after UVB exposure. Notably, the absence of LC in the skin and the depletion of neutrophils at the time point of irradiation prevented a subsequent epidermal thickening as well as cell infiltration upon irradiation (Fig. 4G and H).

In conclusion, skin‐resident LC are important for regulating chemokine response in skin upon UVB exposure leading to neutrophil trafficking which plays an essential role in the induction of apoptosis of DNA‐damaged KC.

## Discussion

Our study presents novel insights into the early events following exposure to high‐dose UVB in the skin. Skin irradiation‐induced DNA damage and apoptosis in KC, as evidenced by CPD formation and the appearance of sunburn cells. The absence of LC in the skin led to a substantial reduction in TNF‐α mRNA levels and diminishing apoptosis induction in damaged KC, thereby leading to an accumulation of DNA‐damaged KC and potentially elevating the risk of developing skin cancer. Our investigation emphasizes the essential involvement of LC in initiating the inflammatory response caused by UVB exposure to the skin.

Programmed cell death upon irreversible DNA damage is required to prevent the introduction of mutations and subsequent carcinogenic effects upon UVB irradiation [36]. The early immunological events directly in the skin after UVB irradiation are still incompletely understood as prior investigations have concentrated on immune responses triggered in skin‐draining lymph nodes [16, 18, 37, 38, 39, 40, 41]. The objective of this study was to examine the involvement of immune cells residing in the skin tissue immediately after the induction of damage through a single high dose of UVB radiation. Our results found that one session of intense UVB‐irradiation‐induced DNA damage in KC, evidenced by the formation of CPD and the subsequent apoptosis of DNA‐damaged KC, represented by the presence of SBC. The further examination of XPA protein mRNA expression levels, a key player in the nucleotide excision repair pathway, showed comparable levels in both irradiated and untreated skin, suggesting a shift toward apoptosis of DNA‐damaged KC rather than active repair. Despite the substantial DNA damage, the repair mechanism appeared limited, which is consistent with earlier investigations using lower UVB doses [28, 29]. The upregulation of the apoptosis‐associated gene Bax, along with the detection of act‐Cas3 further supported the induction of apoptosis in KC following intense UVB exposure.

UV irradiation induces the production of pro‐inflammatory cytokines such as IL‐1 and TNF‐α by KC, dermal fibroblasts, and other inflammatory cells [42, 43]. TNF‐α plays a crucial role as a pro‐inflammatory cytokine, contributing significantly to both innate and adaptive immune responses. Various cell types including macrophages, T cells, mast cells, granulocytes, natural killer (NK) cells, DC, and, as demonstrated by our group and others, LC [32, 44], are capable of producing TNF‐α [45, 46]. Moreover, the blockade of TNF‐α in mice inhibits UVB‐induced epidermal and dermal thickening and the recruitment of inflammatory cells into the dermis [42]. Our exploration of the role of TNF‐α in apoptosis induction of DNA‐damaged KC emphasized its essential role in skin protection. The results obtained from our analysis demonstrated a significant increase in TNF‐α mRNA expression levels post‐UVB irradiation, with TNF‐α neutralization leading to a substantial reduction in act‐Cas3^+^ KC and a decline in SBC numbers. These findings align with prior studies confirming TNF‐α as a key player in apoptosis induction in KC in response to intense UVB irradiation [9, 11].

Work over the last decades has demonstrated that exposure to UVB can induce an immunosuppressive environment [47]. UVB can dose‐ and time‐dependently disturb skin homeostasis [48] due to the introduction of DNA damage and through local and systemic immune suppression which facilitates skin cancer development and infections [49, 50]. Furthermore, UVB leads to the production of cytokines, which can impair LC antigen‐presenting functions [49]. Earlier work revealed that cytokine production in irradiated skin and LC emigration is dependent on the source of UVR as well as the irradiation dose [20, 51, 52]. UVB‐induced LC migration and immunosuppression have been widely studied [16]. However, very few studies focused on the role of LC directly in UVB‐treated skin. These studies made use of Langerin‐DTA mice, which lack LC throughout life [22]. One study claimed a pro‐carcinogenic role for LC in the UVB setting [20], while another stated LC to be protective against UVB‐induced apoptosis in KC [21]. Both studies require careful consideration as the absence of a particular cell type in the skin throughout an individual's lifespan leads to a fundamentally altered immune network among resident skin cells [23]. By utilizing a mouse model, that enables the conditional ablation of LC (huLangerinDTR mice) [53], we examined the role of LC in the skin's response to high‐dose UVB exposure. We found a high proportion of activated LC still being present in the skin 24 h after irradiation, with a decline in their numbers observed after 96 h, suggesting their involvement in the early stages of UVB‐induced skin damage. Skin lacking LC presented with a significant decrease in TNF‐α mRNA expression levels, fewer act‐Cas3^+^ KC and reduced SBC formation, emphasizing the importance of LC in TNF‐α‐mediated apoptosis induction of DNA‐damaged KC following high‐dose UVB exposure. Subsequently, in the absence of LC, DNA‐damaged KC accumulated in the epidermis enhancing the risk of developing non‐melanoma and melanoma skin cancer by increasing numbers of mutations and the risk of cell transformation [54, 55].

To elaborate further, LC were identified as crucial for the chemokine‐mediated recruitment of TNF‐α‐producing neutrophils to UVB‐exposed skin. Depleting LC resulted in a decreased expression of chemokines CXCL‐1 and CXCL‐2, both known to be responsible for neutrophil recruitment [33, 56], leading to a significant decrease in their infiltration. Reduced expression of CXCL‐1 and CXCL‐2 in skin lacking LC suggests their crucial role in regulating chemokine response. The subsequent decrease in neutrophil influx upon LC depletion and the impact on TNF‐α production further emphasize the interconnected nature of these immune responses. Depletion of neutrophils resulted in lower percentages of act‐Cas3^+^ KC and decreased SBC formation, further solidifying the role of neutrophils in the inflammatory response and apoptosis induction. Finally, histological examination revealed a significant increase in epidermal thickness and the presence of cellular infiltrate in skin 48 h after UVB exposure. Notably, the absence of LC in the skin as well as the depletion of neutrophils at the time point of irradiation prevented a subsequent epidermal thickening as well as cell infiltration upon irradiation.

In conclusion, our study provides a comprehensive understanding of the early events following exposure of the skin to high‐dose UVB. The orchestrated interplay between DNA damage, apoptosis induction, and the involvement of TNF‐α, LC, and neutrophils highlights the complexity of the skin's response to intense UVB irradiation. These findings contribute valuable insights for potential therapeutic strategies targeting the prevention and mitigation of UVB‐induced skin damage. Our results further contribute to a nuanced understanding of the underlying mechanisms involved in UVB‐induced skin pathophysiology.

## Novelty and impact

In our study, Langerhans cells emerge as crucial contributors to the initial inflammatory reaction triggered by UVB exposure to the skin. In the absence of Langerhans cells, there was a significant reduction in UVB‐induced skin inflammation. Consequently, the clearance of DNA‐damaged keratinocytes was compromised leading to an elevated risk of developing skin cancer.

## Conflict of interest

The authors declare no financial or commercial conflict of interest.

## Author contributions

Patrizia Stoitzner and Daniela Ortner developed the research project, designed the experiments, and wrote the manuscript. Daniela Ortner, Christoph H. Tripp, and Helen Strandt performed the experiments and analyzed the data. Daniela Ortner, Christoph H. Tripp, and Helen Strandt received technical assistance from Sarah Spoeck, Athanasios Seretis, Florian Hornsteiner, Sophie Dieckmann, and Matthias Schmuth supported the research with scientific advice. All authors read and approved the final manuscript.

### Peer review

The peer review history for this article is available at https://publons.com/publon/10.1002/eji.202451020

**Abbreviations**
act‐Cas3: active caspase‐3
CPDs: cyclobutane pyrimidine dimers
DT: diphtheria toxin
KC: keratinocytes
LN: lymph nodes
SBC: sunburn cell
UV: ultraviolet
UVR: UV radiation
XPA: Xeroderma Pigmentosum group A

## Supporting information



Supporting information

## Data Availability

The data that support the findings of this study are available from the corresponding author upon reasonable request.
