# Supplementary material for: Langerhans cells orchestrate apoptosis of DNA‐damaged keratinocytes upon high‐dose UVB skin exposure
Source: Eur J Immunol. 2024 Sep 17;54(12):2451020. doi: 10.1002/eji.202451020 (PMC11628926; doi:10.1002/eji.202451020)
Supplement: Supplementary file 1 — Supporting information [file EJI-54-2451020-s001.pdf]

Supplemental material

Supplementary Table S1. Anti-mouse antibodies used for flow cytometry

Antibody	Clone	Company
CD207 Alexa 488	929F3.01	Dendritics, Lyon, France
CD11b PerCP-Cy5.5	M1/70	BD Biosciences
CD11c PE-Cy7	N418	Biolegend
CD103 APC	2E7	Biolegend
Ly6G APC	RB6-8C5	Biolegend
CD45 BV510	30-F11	Biolegend
CD3 PE	17A2	Biolegend
CD86 PerCp Cy5.5	GL-1	Biolegend
CD64 PeCy7	X54-5/7.1	Biolegend
MHC II BV421	M5/114.15.2	Biolegend
XCR1 BV650	ZET	Biolegend
CD11b BV605	M1/70	BD Biosciences
MerTK PE	DS5MMER	eBioscience (1, 2)
anti-active caspase-3 PE		BD Biosciences

1. Tamoutounour S, Guilliams M, Montanana Sanchis F, Liu H, Terhorst D, Malosse C, Pollet E, Ardouin L, Luche H, Sanchez C, Dalod M, Malissen B, Henri S. 2013. Origins and functional specialization of macrophages and of conventional and monocyte-derived dendritic cells in mouse skin. *Immunity* 39: 925-38
2. Hornsteiner F, Sykora MM, Tripp CH, Sopper S, Stoitzner P. 2022. Mouse dendritic cells and other myeloid subtypes in healthy lymph nodes and skin: 26-Color flow cytometry panel for immune phenotyping. *Eur J Immunol* 52: 2006-9

Supplementary Table S2. Primers used for RT-qPCR

Gene id/protein	Code or sequence	Company
TATA-binding protein	Forward: ACTTCGTGCAAGAAATGCTGAA Reverse: TGTCCGTGGCTCTCTTATTCTCA	Microsynth AG
Tnf/TNFa	Mm00443258_m1	Thermo Fisher Scientific Inc.
Cxcl1	Mm04207460_m1	Thermo Fisher Scientific Inc.
Cxcl2	Mm00436450_m1	Thermo Fisher Scientific Inc.
Bax	Mm00432051_m1	Thermo Fisher Scientific Inc.
XPA	Mm00457111_m1	Thermo Fisher Scientific Inc.

Supplementary Table S3. Specific markers which were used to define the various cell types by flow cytometric analysis.

**Cell type**	**specific markers**
Langerhans cells	$CD45^+$ /$CD11c^+$ /$CD207^+$
Neutrophils	$CD45^+$ /$CD11b^+$ /$Ly6G^{hi}$
DC	$CD45^+$ /$MHCII^+$/$CD11c^+$
DETC	$CD45^+$ /$CD3^{hi}$ /γδ TCR^+
Macrophages	$CD45^+$ /$CD11b^+$ /$MerTK^+$

Supplementary Figure

Supplementary Figure S1.

A

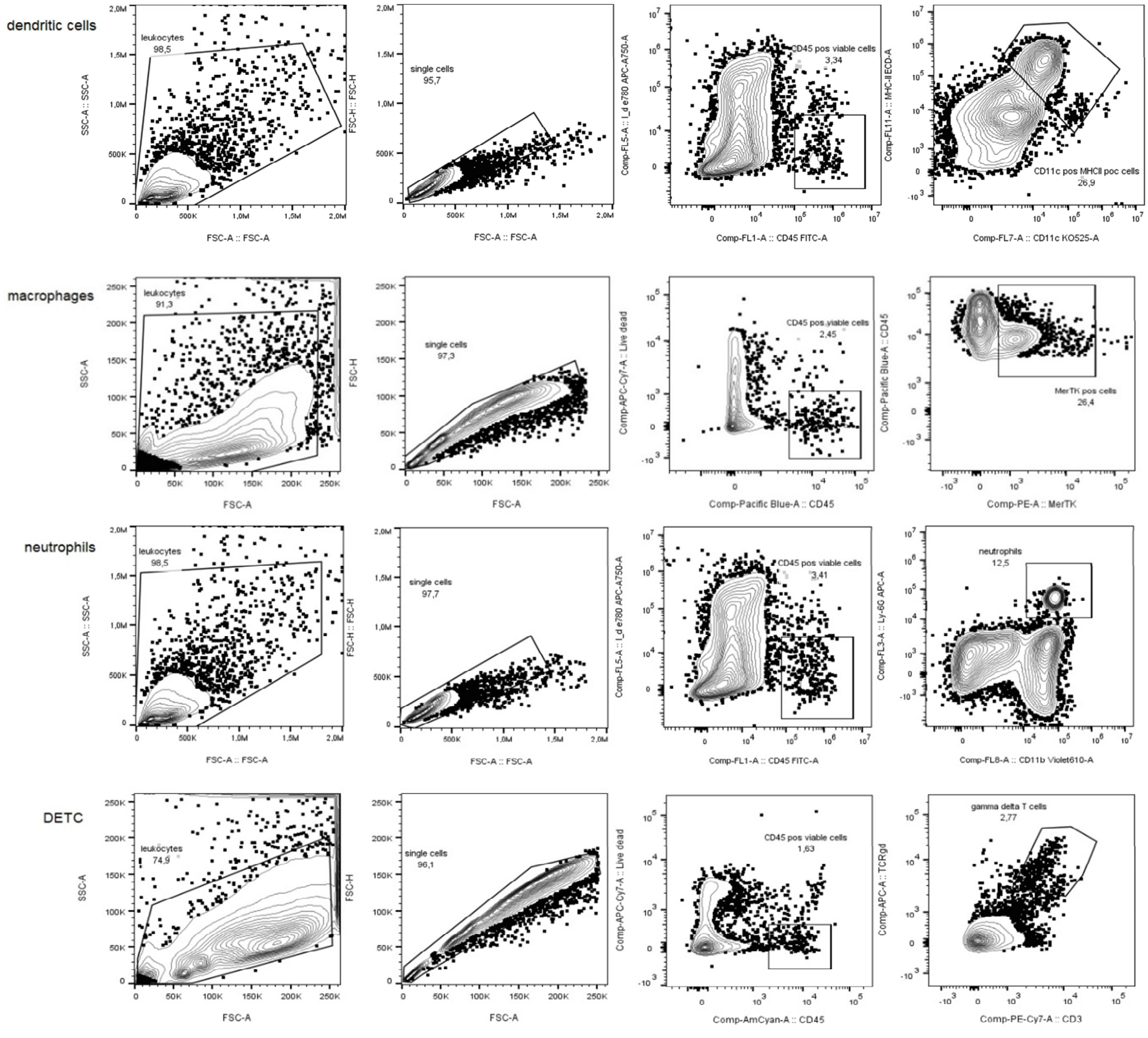

B

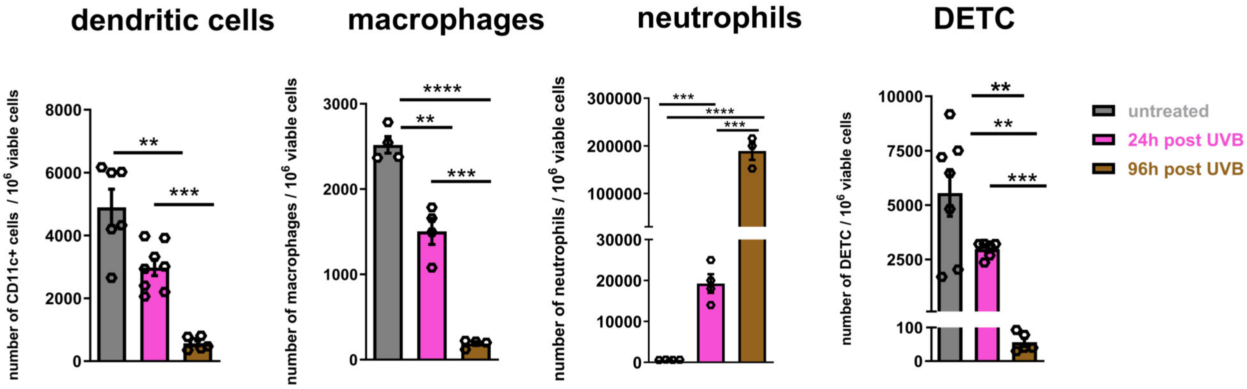

Supplementary Figure S1. Changes in various cell types in UVB-treated skin. Analysis of CD11c$^+$ DC, macrophages, neutrophils and DETC in untreated versus UVB irradiated skin of C57BL/6 mice 24h and 96h after treatment. A) Gating strategy used for the identification of the different cell types in skin cell suspensions. Antibodies are listed in Supplementary Table S3. Cells were pregated to include only viable, single cells. B) Summary graphs display numbers of CD45$^+$MHCII$^+$CD11c$^+$ DC, CD45$^+$CD11b$^+$MerTK$^+$ macrophages, CD45$^+$CD11b$^+$Ly6G^{hi} neutrophils and CD45$^+$CD3^{hi}γδ TCR$^+$DETC in untreated versus UVB-treated skin; mean ± SEM, each data point represents an individual mouse, 3 - 7 mice per group from two independent experiments is shown; *p<0.05; **p<0.01; ***p<0.001; unpaired t-test.
